# Frequency Response and Material Property Sensitivity Analysis of Moving-Coil Geophone Using Finite Element Simulation

**DOI:** 10.3390/s25041008

**Published:** 2025-02-08

**Authors:** Zesheng Yang, Qingfeng Xue, Yi Yao, Yibo Wang

**Affiliations:** 1 State Key Laboratory of Deep Petroleum Intelligent Exploration and Development, Institute of Geology and Geophysics, Chinese Academy of Sciences, Beijing 100029, China; yangzesheng@mail.iggcas.ac.cn (Z.Y.); yiyao16@mail.iggcas.ac.cn (Y.Y.); wangyibo@mail.iggcas.ac.cn (Y.W.); 2 Innovation Academy for Earth Science, Chinese Academy of Sciences, Beijing 100029, China; 3 College of Earth and Planetary Sciences, University of Chinese Academy of Sciences, Beijing 100049, China

**Keywords:** moving-coil geophone, finite element simulation, natural frequency, spurious frequency, frequency response

## Abstract

In the process of unconventional oil and gas production, a large number of microseismic signals are generated. These signals are received by geophones deployed on the ground or in wells and used for safety monitoring. The moving-coil geophone is a commonly used geophone, which is widely used for collecting vibration signals. However, the current conventional moving-coil geophones have certain limitations in terms of frequency band range and cannot fully meet the low-frequency requirements of microseismic signals. We studied the structure and material properties of moving-coil geophones to understand the factors that affect their frequency band. In this paper, we use finite element analysis method to perform structural analysis on a 10 Hz moving-coil geophone, and we combine modal analysis and excitation response analysis to obtain its operating frequency range of 10.63–200.68 Hz. We then discuss the effect of the vibrating components of a moving-coil geophone on its operating frequency range. The material properties of the spring sheet mainly affect the natural frequency of the first-order mode (natural frequency, the lower limit of the operating frequency of the geophone), and the material properties of the lead spring mainly affect the natural frequency of the second-order mode (spurious frequency, the upper limit of the operating frequency of the geophone). By analyzing the sensitivity of the material properties of the vibration system parts and selecting more suitable spring sheets and lead spring materials, a lower natural frequency and a higher spurious frequency can be obtained, thereby achieving the purpose of broadening the operating frequency range of the geophone, which is expected to provide help in actual production.

## 1. Introduction

Unconventional oil and gas resources have attracted widespread attention in recent years, and their exploration and exploitation processes are inevitably accompanied by the generation of a large number of microseismic and induced seismic events. Microseismic monitoring refers to a method used in the unconventional oil and gas extraction process, namely artificial fracturing. Through fracturing operations, rocks break and trigger micro-earthquakes. These microseismic signals will be received by geophones underground or on the ground and then used for research such as identification, location, and source mechanism inversion of microseismic events [1]. Microseismic and induced seismic events usually contain more low-frequency signals, thus placing higher requirements on the frequency band range of the geophone.

A geophone is a sensor used to detect and record ground shaking. In seismology, the propagation characteristics of seismic waves detected by seismometers can provide insights into the geological structure of the Earth’s interior. According to different working principles, geophones can be divided into different types, such as moving coil, capacitive, piezoelectric, electrochemical, and MEMS. At present, the mainstream acquisition probes mostly use moving-coil geophones [2,3]. Moving-coil geophones have the advantages of having a low cost, simple structure, and stable technical performance; being self-powered; and having the ability to easily form a node network [4,5,6]. Although the moving-coil geophone is a relatively early type used in earthquake monitoring, it still has broad development prospects in the field of seismology. Moving-coil geophones work on the principle of Faraday electromagnetic induction. When the ground vibrates, the coil inside the geophone moves in the magnetic field, generating an induced voltage. The vibration velocity of the ground can be calculated through electromechanical equations. Moving-coil geophones are widely used in active source seismic exploration, background noise analysis, urban underground structure monitoring, coal mining, and tunnel-drilling safety monitoring [7,8,9].

However, since the moving-coil geophone has low sensitivity at low frequencies, this limits the lower frequency band of the moving-coil geophone [10]. In order to expand the frequency band of the moving-coil geophone, the method of selecting more suitable component materials or optimizing the structure of the geophone is often adopted. As a effective numerical calculation method, finite element analysis has been widely used in simulation in various physical fields. Using finite element analysis method to study the structural mechanics and electromagnetic field distribution of moving-coil geophones helps to give moving-coil geophones a wider frequency band and higher sensitivity [11,12,13,14]. By establishing the corresponding mechanical model, the displacement response of the object under the action of external force can be effectively analyzed, and an effective method and theoretical basis can be provided for further studying its mechanical properties, such as strength and stiffness. Through the quantitative analysis of the displacement response, the safety and stability of the object under various working conditions can be more accurately evaluated, providing an important reference for structural design, material selection, and optimization [15,16]. As a part of the vibration system of the moving-coil geophone, the spring sheet can obtain the required working frequency band by optimizing the structure of the spring sheet and selecting a more suitable spring material [17,18]. At present, there are also studies on widening the frequency band by designing corresponding compensation circuits without changing the material and structure of the geophone. Among them, low-frequency compensation circuits such as force balance feedback circuits and zero-pole circuits have achieved good application results [19,20,21,22,23,24].

In this paper, we simulated a 10 Hz moving-coil geophone by the finite element method. Through modal analysis and excitation response analysis, we determined the operating frequency range of the geophone. Finite element simulation results show that the operating frequency range of the seismic sensor is from 10.63 Hz to 200.68 Hz, which has a smaller error range compared to the range from 10 Hz to 200 Hz in the sensor parameter indicators. In the Discussion section of the paper, we analyze the material properties of the parts inside the geophone that have relative motion with the outer shell and discuss how we tested the effects of density, ρ; Young’s modulus, E; and Poisson’s ratio, μ, on the modal frequency. The material properties of the spring sheet have a greater influence on the natural frequency of the first-order mode. As the density and Poisson’s ratio increase, or the Young’s modulus decreases, the natural frequency of the first-order mode decreases. The material properties of the lead spring have a greater influence on the natural frequency of the second-order mode. As the density and Poisson’s ratio decrease, or the Young’s modulus increases, the natural frequency (spurious frequency) of the second-order mode increases. Thus, we have obtained the influence of the material properties of the spring sheet and the lead spring on the working frequency band of the moving-coil geophone. In actual production, we can select the spring sheet material with a higher density, smaller Young’s modulus, and larger Poisson’s ratio, and the lead spring material with a lower density, larger Young’s modulus, and smaller Poisson’s ratio, so as to achieve the purpose of reducing the natural frequency, increasing the spurious frequency and expanding the frequency band range of the moving-coil geophone.

## 2. Moving-Coil Geophone

### 2.1. Working Principle of the Moving-Coil Geophone

**Figure 1:**
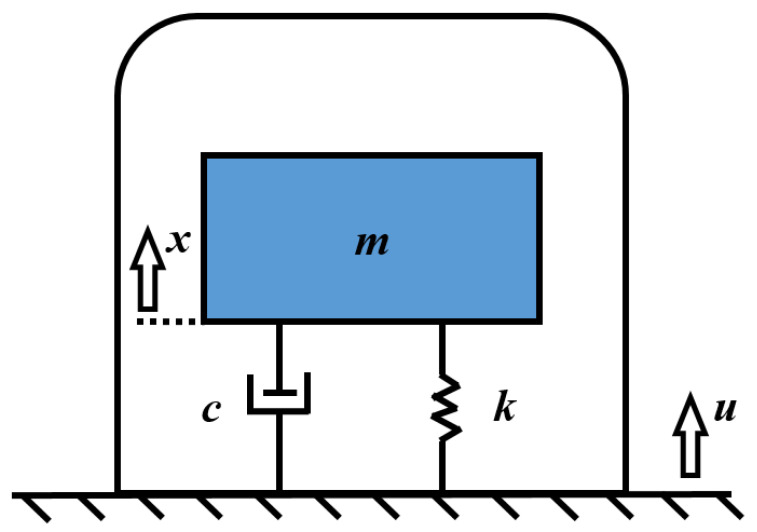
Simplified diagram of the working principle of a moving-coil geophone.

The working principle of the moving-coil geophone is that when the geophone is coupled to the ground surface, and when the ground surface vibrates, the inertial body (composed of a coil and a coil frame) inside the geophone will have the inertia to remain stationary because it is connected to the outer shell through a spring sheet. The coil contained in the inertial body will move axially relative to the magnet and cut the magnetic flux lines at the same time. According to Faraday’s law of electromagnetic induction, the coil will generate an induced voltage and output a voltage signal. As shown in Figure 1, the mechanical equation of the moving-coil geophone is as follows [25,26,27]:

(1) $$m\ddot{x}+c\left(\dot{x}-\dot{\;u}\right)+k\left(x-u\right)=0,$$

In Formula (1), m is the mass of the inertial body; u is the displacement of the ground; x is the displacement of the inertial body; c is the damping coefficient; k is the spring stiffness; and G is the open-loop sensitivity parameter of the geophone, where G = Bl, B is the effective magnetic field intensity, and l is the total length of the coil.

The transfer function *H*(*jω*) of a moving-coil geophone can be expressed as the ratio of the output voltage to the ground vibration velocity:

(2) $$H\left(j\omega \right)=\frac{U_{0}}{V_{0}}=\frac{-Gs^{2}}{s^{2}+2h_{0}\omega_{0}s+{\omega_{0}}^{2}},$$

(3) $$h_{0}=\frac{c+\frac{G^{2}}{R}}{2m\omega_{0}},$$

(4) $$\omega_{0}=\sqrt{\frac{k}{m}},$$

In Formula (2), *U*_0_ is the voltage output value, *V*_0_ is the vibration velocity of the ground, R is the circuit resistance, ω is the angular frequency, *ω*_0_ is the natural frequency, j is an imaginary number, and *h*_0_ is the damping ratio.

### 2.2. Main Technical Indicators of Moving-Coil Geophone

The natural frequency of a moving-coil geophone is also called the inherent frequency. It is the external excitation frequency that causes the axial vibration resonance of the geophone and is completely determined by the properties of the object itself. As one of the technical indicators of the moving-coil geophone, the natural frequency affects the lower limit of the geophone’s frequency band. When the system damping is not considered, the natural frequency, *f*_0_, is as follows:

(5) $$f_{0}=\frac{1}{2\pi }\sqrt{\frac{k}{m}},$$

In Formula (5), we can see that the natural frequency is related to the mass and stiffness of the object itself. The larger the mass and the smaller the stiffness, the lower the natural frequency. However, for the geophone, this will increase the size of the geophone, and the spring will be easily damaged. Therefore, it is necessary to comprehensively consider the characteristics of the structure itself to achieve the purpose of reducing the natural frequency.

On the other hand, aliasing affects the upper frequency band of the geophone due to the presence of resonant frequencies perpendicular to the spring vibration direction. The natural frequency and the spurious frequency determine the working frequency band of the geophone [28]. Within this frequency band, the sensitivity has good linearity and is approximately a constant.

Sensitivity is a measure of the ability of a moving-coil geophone to receive signals. From the transfer function, we can see that it is the ratio of the voltage value output by the moving-coil geophone to the received vibration velocity. The greater the sensitivity, the stronger the ability to receive tiny earthquakes. Therefore, it is one of the most important technical indicators of a moving-coil geophone [14,29].

In this paper, we mainly study the effective working frequency band of the moving-coil geophone structure, without considering technical indicators such as sensitivity. The effective working frequency band of the moving-coil geophone is determined by the natural frequency and the spurious frequency. In order to expand its frequency band, it is hoped to have a lower natural frequency and a higher spurious frequency.

## 3. Finite Element Simulation

In order to deeply analyze the working frequency band of the moving-coil geophone, we simulated a 10 Hz moving-coil geophone. Finite element simulation can be roughly divided into three parts: pre-processing, solution and post-processing [30,31,32]. Finite element simulation can be roughly divided into three parts: pre-processing, solving, and post-processing. The pre-processing stage includes establishing a simulation model or importing a geometric model, defining material properties, and performing finite element meshing. The solving stage covers fixed constraints and contact settings, force load settings, and calculation analysis. Finally, the post-processing stage includes mode and vibration shape extraction; displacement and deformation result display; and other analysis result extractions, such as static analysis, explicit dynamics analysis, etc. [33,34].

**Figure 2:**
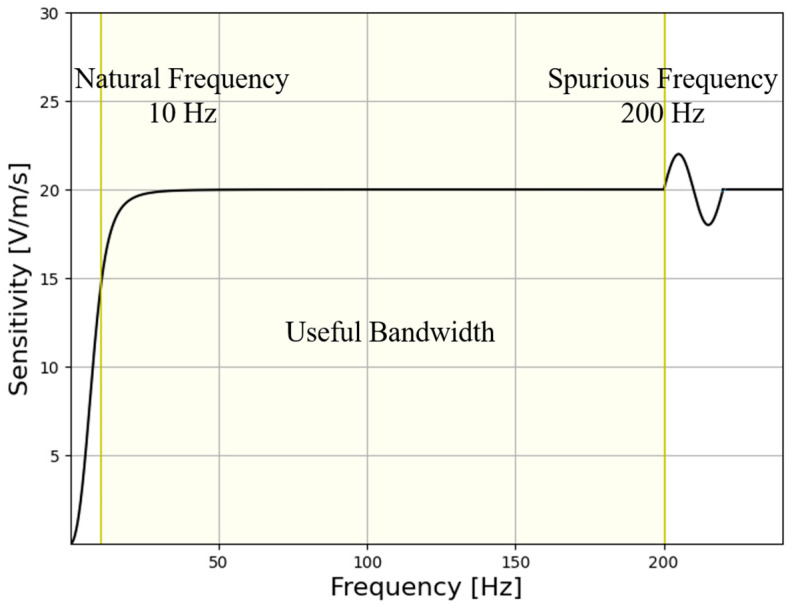
Frequency-response curve of 10 Hz geophone.

As shown in Table 1, the main technical indicators of the 10 Hz moving-coil geophone used in this paper are shown. According to the introduction in Section 2.2, its effective operating frequency range is 10–200 Hz. Figure 2 is the frequency response curve of the geophone, with aliasing occurring at 200 Hz.

### 3.1. Establishment of Finite Element Model

**Figure 3:**
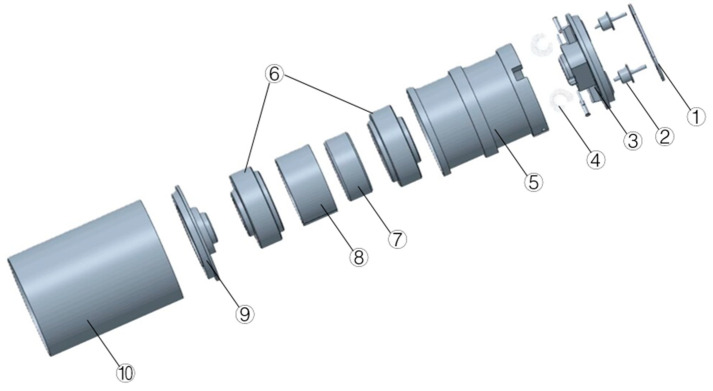
Exploded view of the moving-coil geophone geometric model: ① insulating gasket; ② insulator; ③ top cover and terminal; ④ lead spring; ⑤ coil frame; ⑥ magnetic shoe; ⑦ magnetic steel; ⑧ compensation ring; ⑨ bottom cover; and ⑩ shell.

Firstly, we established the geometric model of 10 Hz moving-coil geophone. Figure 3 is an exploded view of the moving-coil geophone parts, where we can see all the parts in the geophone. The outer shell, top cover, bottom cover, insulating gasket, and insulator constitute the external structure; the spring sheet, coil frame, and coil together constitute the vibration system in the geophone; and the magnet, magnetic shoe, and compensation ring constitute the magnetic system assembly. The spring sheet, coil frames, and coil, which are the vibration components, are the main parts we analyze. In the description of Section 2.1, these components will experience relative displacement with other components, affecting the frequency band range of the geophone. We then performed pre-processing, including setting material properties and meshing.

**Figure 4:**
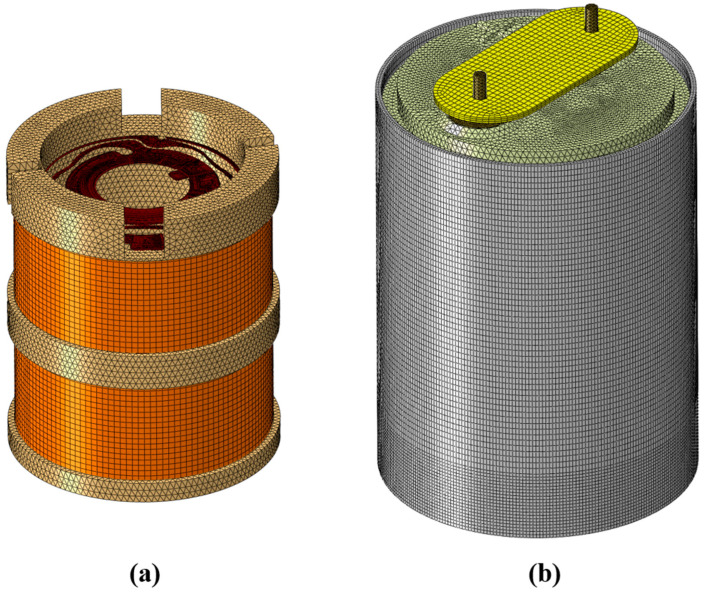
Grid division of moving-coil geophone. (**a**) Internal structure of the geophone. (**b**) External structure of the geophone.

Figure 4a shows the meshing of the moving-coil geophone, where the coil frame, spring sheet, and other parts can be seen. Figure 4b shows the meshing of the encapsulated geophone with a housing, with 931,027 nodes and 1,799,413 elements.

The material properties of the moving-coil geophone are shown in Table 2. The most commonly used material for the spring sheet is beryllium copper, which is an ideal high-strength elastic material and has the characteristics of being non-magnetic, wear-resistant, corrosion-resistant, fatigue-resistant, and stress relaxation-resistant. The production process of spring leaves made of beryllium bronze is very mature in the market. The density of beryllium bronze used in this article is 8.3 × 10^−9^ t/mm^3^, Young’s modulus is 105,140 MPa, and Poisson’s ratio is 0.3 [35]. Since the coil is tightly wound on the coil frame, it is difficult to model it with the actual thickness of the coil. We will equate it to a ring wrapped around the coil frame. The mass of the coil frame and the coil is 11 g, of which the mass of the coil frame is 4 g. The volume of the ring is calculated based on the density of the coil [36]. As shown in Figure 4a, the light yellow part is the coil frame, and the orange part is the same coil tightly wound in the upper and lower grooves of the coil frame. The coils in the upper and lower parts are wound in opposite directions with the same number of turns, and the current direction generated when cutting the magnetic flux lines is the same [14]. The two ends of the coil are connected to the lead springs, which are then connected to the terminals and insulators to output the generated induced current to the external circuit. In this paper, we do not consider electromagnetic phenomena such as electromagnetic induction caused by magnetic components. We mainly analyze the structure of the moving-coil geophone and study the characteristics of its structure itself.

**Figure 5:**
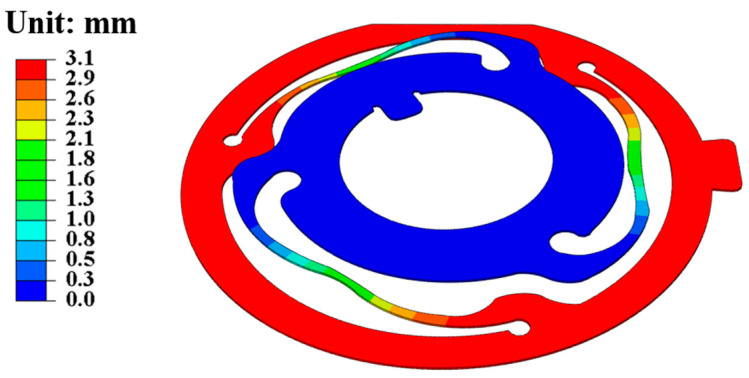
Schematic diagram of the deformation of the upper spring leaf.

In the contact condition setting, since the geophone housing, top cover, magnetic shoe, magnetic steel, compensation ring, and bottom cover are compacted and fixed in actual assembly, they are set to fixed binding contact. The coil frame, coil, and spring sheet are used as a vibration system in which the inner ring of the spring sheet and the top cover are in fixed binding contact, the outer ring of the spring sheet and the coil frame are in fixed binding contact, and the inner and outer rings of the spring sheet are connected by spring arms. When the external environment vibrates, the coil frame and the housing will produce relative displacement, and the spring arm will deform. Figure 5 offers a schematic diagram of the deformation of the spring sheet, showing that the displacement of the inner ring of the spring sheet is 0, and the displacement of the outer ring of the spring sheet is the maximum.

Boundary conditions are very important for modal analysis, as they can affect the mode shapes and natural frequencies of a structure. In the boundary condition setting, the bottom surface of the geophone is used as a fixed constraint, the six degrees of freedom of the bottom cover of the geophone are fixed, the coupling between the bottom surface of the geophone and the ground is simulated, and the constraint mode is calculated to make the constraint meet the actual physical situation. If there is no constraint, the calculated mode is an ideal free mode, the model has six rigid body motion directions, and the natural frequencies of the 1st-to-6th-order modes are basically 0. Under this constraint, the inner coil former will have relative motion with the outer shell.

### 3.2. Modal Analysis

Modal analysis is the basis of all structural dynamics analyses. It can obtain the vibration characteristics of the structure, including the national frequency, modal vibration shape, etc., so as to avoid resonance of the structure. The material parameters required for modal analysis generally include density, Young’s modulus, and Poisson’s ratio. The mode depends on the inherent properties of the structure itself, and its mode will not change regardless of the size of the external load. When the geometric dimensions or contact relationship of the structure is changed, the natural frequency and vibration mode will change accordingly. Under given contact conditions, modal analysis can obtain modal frequency values and vibration type displacement cloud diagrams, thereby guiding the design to avoid these resonant frequency points. Each natural frequency of the structure corresponds to a different vibration mode. In our study, the natural frequency is an important characteristic parameter that needs to be paid attention to. The natural frequencies introduced in Section 2.2 correspond to the natural frequencies of the first-order mode, and the aliased frequencies appear corresponding to the natural frequencies of the second-order mode. Therefore, through modal analysis, we can obtain the effective operating frequency band of the moving-coil geophone.

**Figure 6:**
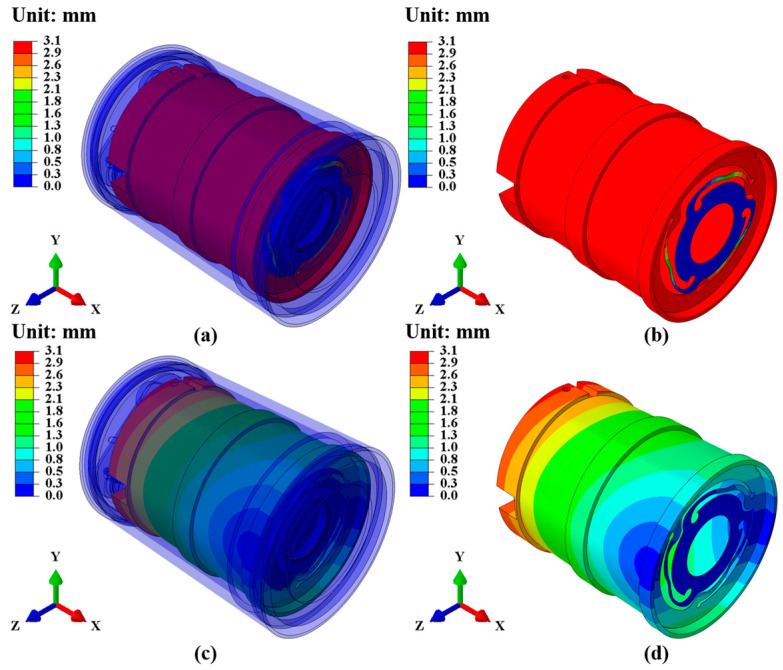
First-order and second-order modal displacement cloud diagrams of moving-coil geophones, in mm, with X being the vertical direction, and Y and Z being the horizontal directions. (**a**) The first-order modal displacement cloud diagram of the geophone as a whole. (**b**) The first-order modal displacement cloud diagram of the coil frame and spring sheet. (**c**) The second-order modal displacement cloud diagram of the geophone as a whole. (**d**) The second-order modal displacement cloud diagram of the coil frame and spring sheet.

The results of the extracted modal analysis show that the natural frequency of the first-order mode is 10.63 Hz, and that of the second-order mode is 200.68 Hz, as shown in Table 3. In Section 3.1, “Contact Settings and Constraints”, we constrained the bottom cover to 6 degrees of freedom and set it to bound contact with parts such as the outer shell. Therefore, the coil frame and spring leaf act as a vibration system and move relative to the outer shell. The vibration shape of the first-order mode is the natural vibration motion perpendicular to the ground, as shown in Figure 6a,b; the vibration shape of the second-order mode is the natural vibration motion in the horizontal direction, as shown in Figure 6c,d. The generation of aliasing is because the moving-coil geophone will resonate at its natural frequency, affecting the reception of ground vibration signals. The resonance generated after the second-order mode can be considered as the influence of aliasing. The natural frequencies of the first two modes affect the effective working frequency band of the moving-coil geophone [18,37]. According to the extracted modal analysis, the effective operating frequency band of the moving-coil geophone is 10.63–200.68 Hz.

### 3.3. Stimulus Response Analysis

In this section, we use excitation response analysis to simulate the response of a moving-coil geophone when it is subjected to ground vibration. Excitation response analysis is an important method for studying the dynamic response of a structure. By loading excitation and combining it with frequency domain analysis, the response of the structure is studied in the frequency domain to obtain the frequency response of the structure. We can analyze the frequency response curve to obtain the effective operating frequency band of the geophone.

**Figure 7:**
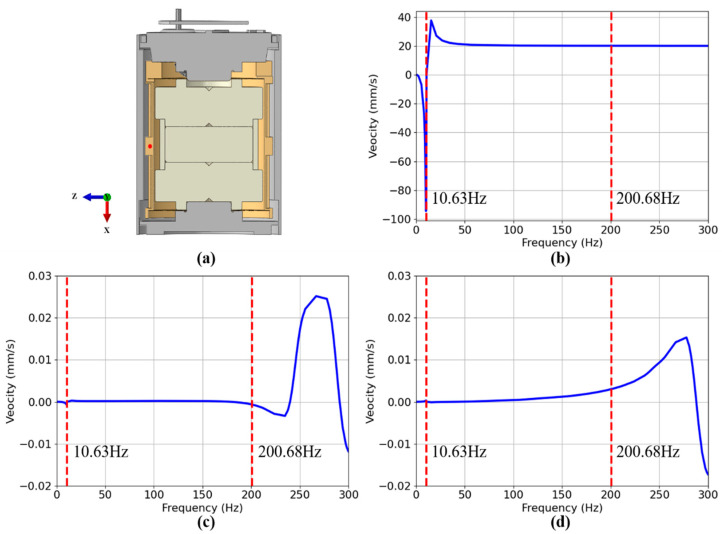
Excitation-response curve of monving coil geophone 0–300 Hz. (**a**) Cross-section of a moving-coil geophone, with the red points taken for frequency-response analysis in three directions. (**b**) Velocity response in the vertical X direction. (**c**) Velocity response in the horizontal Y direction. (**d**) Velocity response in the horizontal Z direction.

To simulate the response of the moving-coil geophone when receiving ground vibration, we applied a vertical excitation at the bottom of the geophone with a velocity of 1 mm/s. A point on the coil frame is taken for analysis, as shown in the red point in Figure 7a. The vertical and two horizontal excitation response curves are drawn at this point in the frequency range of 0–300 Hz. It can be seen from Table 3 that aliasing occurs within 0–300 Hz. In Figure 7b, there is a resonant response in the vertical direction at the natural frequency of the first-order mode, with a maximum speed of 94.38 mm/s, and the direction is vertically downward. The response at the natural frequency of the second-order mode is relatively weak; in Figure 7c,d, there is a resonant response in the horizontal direction at the natural frequency of the first-order mode, and the resonant response is more obvious after the natural frequency of the second-order mode. The finite element model of a moving-coil geophone has a flat response between the natural frequencies of the first and second modes that is generally considered to be the effective operating frequency band [28]. The natural frequency of the second-order mode affects the transverse resonance of the geophone, causing the received vibration signal to be distorted, that is, spurious frequency. In the effective working frequency band, the response of the moving-coil geophone to external excitation is stable, and the corresponding sensitivity has good linearity, which is approximately a constant, which is what we need in seismic exploration.

## 4. Discussion

After finite element simulation of the moving-coil geophone, we realized the relationship between the natural frequency of the first and second modes and the operating frequency band. As microseismic exploration has received more and more attention from researchers, it has wide applicability in engineering projects such as unconventional oil and gas exploration, and urban underground structure exploration. Conventional moving-coil geophones have low sensitivity at low frequencies. In order to receive the lower-frequency signals required for microseismic exploration, it is necessary to expand the frequency band of the moving-coil geophone. Since the coil frame, coil, and spring sheet are part of the vibration system in the moving-coil geophone, we analyzed the sensitivity of these parts to the operating frequency band and tested the effects of density, ρ; Young’s modulus, E; and Poisson’s ratio, μ, on the modal frequency to find suitable material properties.

The natural frequency of the first-order mode of the moving-coil geophone corresponds to Equation (5). The change in the density of the spring sheet actually affects the change in the mass, m, in Equation (5), thereby changing the natural frequency of the first-order mode, that is, the natural frequency. As shown in Table 4, the density of the spring sheet increases, and the natural frequency decreases. As shown in Table 5, the Young’s modulus of the spring sheet increases, and the natural frequency increases. As shown in Table 6, the Poisson’s ratio of the spring sheet increases, and the natural frequency decreases.

**Figure 8:**
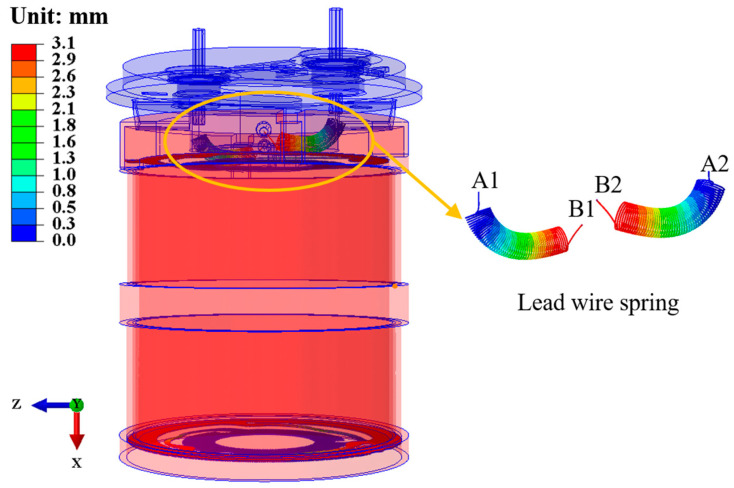
Schematic diagram of the lead spring of the moving-coil geophone under the first-order-mode vibration shape.

In the modal analysis, we found that the material properties of the lead spring have a great influence on the vibration mode and natural frequency of the second-order mode. The changes in the density, ρ; Young’s modulus, E; and Poisson’s ratio, μ, of the lead spring have a great influence on the second-order mode. As shown in Figure 8, the A1 end and the A2 end are connected to the insulator, where the insulator is connected to the external circuit; the B1 end and the B2 end are connected to the terminal, where the terminal is connected to the coil. Therefore, the role of the lead spring in the moving-coil geophone is to output the induced current generated by the coil to the external circuit. In Figure 8, the displacement of the A1 end and the A2 end is small, almost 0; the B1 end and the B2 end are equivalent to pulling the coil and the coil frame, and the displacement is the largest as the coil and the coil frame vibrate. In principle, the lead spring does not participate in the vibration of the coil frame and the spring sheet. The lead spring cannot generate tension or pressure on the vibration system, and it is freely connected to the coil and the top cover assembly on the coil frame. Under ideal conditions, the elastic coefficient of the lead spring will not affect the vibration of the moving-coil geophone. However, in actual tests, the elastic coefficient of the lead spring has a significant impact on the amplitude–frequency curve of the moving-coil geophone [38].

By changing the material properties of the lead spring, we found that it is essentially an elastic element and will also participate in the vibration of the vibration system, affecting the mode shape and natural frequency of the moving-coil geophone. Among them, the impact on the second-order mode is greater, which is closely related to the appearance of spurious frequencies. As shown in Table 7, as the density of the lead spring increases, the second-order modal natural frequency decreases; as the Young’s modulus of the lead spring increases, the second-order modal natural frequency increases, as shown in Table 8; and as the Poisson’s ratio of the lead spring increases, the second-order modal natural frequency decreases, as shown in Table 9.

Based on the above simulation results, we can see that the material properties of the spring sheet have a greater impact on the natural frequency of the first-order mode. As the density and Poisson’s ratio increase, or the Young’s modulus decreases, the natural frequency of the first-order mode decreases, while the material properties of the lead spring have a greater impact on the natural frequency of the second-order mode. As the density and Poisson’s ratio increase, or the Young’s modulus decreases, the natural frequency (spurious frequency) of the second-order mode decreases. If we want a wider effective working frequency band, we need a smaller natural frequency for the first-order mode and a larger natural frequency for the second-order mode. Among them, spring sheet materials with a higher density, smaller Young’s modulus, and larger Poisson’s ratio, and lead spring materials with a lower density, larger Young’s modulus, and smaller Poisson’s ratio can be selected, so as to achieve the purpose of reducing the natural frequency, increasing the spurious frequency, and expanding the frequency band range of the moving-coil geophone.

## 5. Conclusions

Under the current demand for microseismic exploration, the moving-coil geophone needs a wider frequency band and can receive lower frequency signals. In order to analyze the factors affecting the frequency band range of the moving-coil geophone, we conducted a study using finite element analysis method. We successively performed geometric model establishment, meshing, material property definition, constraint and contact settings, modal analysis and excitation response. The natural frequencies of the first six modes were obtained through modal analysis, and the operating frequency range of the sensor was determined from the excitation response analysis to be 10.63–200.68 Hz. Among the technical indicators of the moving-coil geophone, the natural frequency is 10 Hz, and the spurious frequency is ≥200 Hz, which is consistent with the finite element analysis results.

We continue to conduct finite element analyses on the geophone itself. The material properties of the spring sheet have a greater influence on the natural frequency of the first-order mode. As the density and Poisson’s ratio increase, or the Young’s modulus decreases, the natural frequency of the first-order mode decreases; meanwhile, the material properties of the lead spring have a greater influence on the natural frequency of the second-order mode. As the density and Poisson’s ratio increase, or the Young’s modulus decreases, the natural frequency (spurious frequency) of the second-order mode decreases.

Based on the relationship obtained from the analysis, we can select more appropriate material properties to achieve the purpose of widening the working frequency band and monitoring microseismic signals. By analyzing the sensitivity of the material properties of vibration system parts, we can optimize the material properties of the moving-coil geophone itself, which is expected to provide help in actual production. Our subsequent optimization can be carried out from the topological optimization of the structure of the moving-coil geophone itself, and new designs can be made on the existing moving-coil geophone structure to change the structural properties, which can also achieve the purpose of widening the frequency band.

## Figures and Tables

**Table 1:** Technical indicators of 10 Hz geophone.

Geophone Technical Indicators (20 °C)	
Natural frequency	10 Hz ± 5%
Sensitivity	20 V/m/s ± 5%
Damping coefficient	0.7 ± 5%
Spurious frequency	≥200 Hz

**Table 2:** Material properties of the main parts of the moving-coil geophone.

Component	Density (t/mm^3^)	Young’s Modulus (MPa)	Poisson’s Ratio	Material
Shell	7.85 × 10^−9^	200,000	0.3	No. 15 steel
Top cover	6.7 × 10^−9^	130,000	0.3	ZA4-1 cast zinc alloy
Bottom cover	6.7 × 10^−9^	130,000	0.3	ZA4-1 cast zinc alloy
Spring sheet	8.3 × 10^−9^	105,140	0.3	Beryllium bronze
Coil frame	2.77 × 10^−9^	71,700	0.33	LY12 aluminum alloy
Coil	8.9 × 10^−9^	110,000	0.37	Enameled wire
Magnetic steel	7.62 × 10^−9^	160,000	0.3	NdFeB
Magnetic shoe	7.85 × 10^−9^	200,000	0.3	No. 15 steel
Compensation ring	7.85 × 10^−9^	210,000	0.3	Steel
Lead spring	7.55 × 10^−9^	160,000	0.3	Beryllium copper wire

**Table 3:** Natural frequencies of 1st-to-6th-order modes.

Modal Order	Natural Frequency (Hz)
First-order mode	10.63
Second-order mode	200.68
Third-order mode	202.07
Fourth-order mode	245.06
Fifth-order mode	245.43
Sixth-order mode	288.03

**Table 4:** Relationship between the density of the spring sheet and the natural frequencies of the first two modes.

Density (t/mm^3^)	First-Order Mode (Hz)	Second-Order Mode (Hz)
8.1 × 10^−9^	10.633	200.68
8.2 × 10^−9^	10.632	200.68
8.3 × 10^−9^	10.631	200.68
8.4 × 10^−9^	10.63	200.68
8.5 × 10^−9^	10.63	200.68
8.6 × 10^−9^	10.629	200.68

**Table 5:** Relationship between the Young’s modulus of the spring and the natural frequencies of the first two modes.

Young’s Modulus (MPa)	First-Order Mode (Hz)	Second-Order Mode (Hz)
100,000	10.376	200.68
105,000	10.624	200.68
105,140	10.631	200.68
110,000	10.867	200.68
115,000	11.105	200.68
120,000	11.338	200.69
125,000	11.565	200.69
130,000	11.789	200.69

**Table 6:** Relationship between the Poisson’s ratio of the spring and the natural frequencies of the first two modes.

Poisson’s Ratio	First-Order Mode (Hz)	Second-Order Mode (Hz)
0.25	10.695	200.68
0.27	10.669	200.68
0.29	10.644	200.68
0.3	10.631	200.68
0.31	10.619	200.68
0.33	10.595	200.68
0.35	10.572	200.68

**Table 7:** Relationship between the density of the lead spring and the natural frequencies of the first two modes.

Density (t/mm^3^)	First-Order Mode (Hz)	Second-Order Mode (Hz)
7.5 × 10^−9^	10.63	201.48
7.55 × 10^−9^	10.631	200.68
8.0 × 10^−9^	10.632	195.09
8.3 × 10^−9^	10.632	191.53

**Table 8:** Relationship between the Young’s modulus of lead spring and the natural frequencies of the first two modes.

Young’s Modulus (MPa)	First-Order Mode (Hz)	Second-Order Mode (Hz)
110,000	10.584	166.53
120,000	10.594	173.93
130,000	10.603	181.03
140,000	10.613	187.86
150,000	10.623	194.44
160,000	10.631	200.68

**Table 9:** Relationship between the Poisson’s ratio of the lead spring and the natural frequencies of the first two modes.

**Poisson’s Ratio**	**First-Order Mode** **(Hz)**	**Second-Order Mode** **(Hz)**
0.25	10.634	201.17
0.27	10.633	200.91
0.29	10.632	200.74
0.3	10.631	200.68
0.31	10.631	200.66
0.33	10.63	200.7
0.35	10.63	200.89

## Data Availability

Data collected through the research presented in the paper are available upon request from the corresponding author.
